# Recent advances in the roles of extracellular vesicles in cardiovascular diseases: pathophysiological mechanisms, biomarkers, and cell-free therapeutic strategy

**DOI:** 10.1186/s10020-025-01200-x

**Published:** 2025-05-05

**Authors:** Mengyang Wang, Yuwu Chen, Biyi Xu, Xinxin Zhu, Junke Mou, Jiani Xie, Ziao Che, Liyang Zuo, Ji Li, Haibo Jia, Bo Yu

**Affiliations:** 1 Department of Cardiology, 2nd Affiliated Hospital of Harbin Medical University, National Key Laboratory of Frigid Zone Cardiovascular Diseases, Harbin, 150001 People’s Republic of China; 2 The Key Laboratory of Myocardial Ischemia, Chinese Ministry of Education, Harbin, 150001 People’s Republic of China; 3 College of Pharmacy, Harbin Medical University, Harbin, 150081 People’s Republic of China

**Keywords:** Cardiovascular diseases, Extracellular vesicles, Exosomes, Cell-free therapy, Intercellular communication

## Abstract

Cardiovascular diseases (CVDs) represent a profound challenge with inflammation playing a significant role in their pathophysiology. Extracellular vesicles (EVs), which are membranous structures encapsulated by a lipid bilayer, are essential for intercellular communication by facilitating the transport of specific bioactive molecules, including microRNAs, proteins, and lipids. Emerging evidence suggests that the regulatory mechanisms governing cardiac resident cells are influenced by EVs, which function as messengers in intercellular communication and thereby contribute to the advancement of CVDs. In this review, we discuss the multifaceted biological functions of EVs and their involvement in the pathogenesis of various CVDs, encompassing myocardial infarction, ischemia–reperfusion injury, heart failure, atherosclerosis, myocarditis, cardiomyopathy, and aneurysm. Furthermore, we summarize the recent advancements in utilizing EVs as non-invasive biomarkers and in cell-free therapy based on EVs for the diagnosis and treatment of CVDs. Future research should investigate effective techniques for the isolation and purification of EVs from body fluids, while also exploring the pathways for the clinical translation of therapy based on EVs. Additionally, it is imperative to identify appropriate EV-miRNA profiles or combinations present in the circulation of patients, which could serve as biomarkers to improve the diagnostic accuracy of CVDs. By synthesizing and integrating recent research findings, this review aims to provide innovative perspectives for the pathogenesis of CVDs and potential therapeutic strategies.

## Introduction

Cardiovascular disease (CVD), recognized as the primary cause of mortality globally, constitutes a prevalent and persistent chronic disease (Vaduganathan et al. 2022). Of note, conditions such as myocardial infarction (MI), ischemia–reperfusion (I/R) injury, heart failure (HF), atherosclerosis (AS), myocarditis, cardiomyopathy, and aneurysm collectively account for a substantial portion of CVD deaths worldwide (Tsao et al. 2023). Although inflammation has been extensively acknowledged as a critical contributor to CVDs over the past few decades (Carnevale 2022), the other underlying molecular mechanisms remain inadequately elucidated.

Extracellular vesicles (EVs), as defined by the Minimal information for studies of extracellular vesicles (MISEV2023) guidelines, are lipid bilayers lacking a functional nucleus, released by various cells (Welsh et al., 2024). It is recognized that EVs contain a diverse array of biomolecules, including proteins, lipids, nucleic acids, and metabolites, which enable them to play significant roles in numerous biological functions (Kalluri and LeBleu 2020). For instance, cell functions can be modulated by EVs as conduits of intercellular communication through the transfer of their cargo to recipient cells, thereby regulating the development and progression of diseases (Marar et al. 2021). EVs are also capable of altering the extracellular matrix by transporting regulatory molecules, consequently facilitating tissue repair and remodeling (Johnson et al. 2023). Furthermore, EVs can be engineered to encapsulate therapeutic agents, such as drugs, proteins, and nucleic acids, for targeted delivery to specific cells or tissues (Lyu et al. 2023). Given their intricate composition and pivotal roles in various intricate biological processes, EVs are of great significance for the initiation and progression of diseases,while also possessing considerable potential as biomarkers for disease diagnosis, strategies for cell-free therapy, and indicators for prognostic evaluation and therapeutic monitoring (Johnson et al., 2023; Petracci et al., 2024; Ricklefs et al., 2024)

In recent years, the significant role of EVs in the pathophysiology and treatment of CVDs has been confirmed by several studies (Lee et al., 2025; Neves et al. 2023; Saheera et al. 2021; Sahoo et al. 2021). For instance, the cyclic GMP-AMP synthase-stimulator of interferon genes (cGAS-STING) pathway can be aberrantly activated by damaged DNA within EVs, thereby exacerbating vascular inflammation and contributing to CVDs such as MI, HF, and aortic aneurysm (Oduro et al. 2022). However, various types of stem cell-derived EVs may have potential therapeutic effects on injured heart (Rezaie et al. 2019). This review summarizes the biogenesis, structure, and classification of EVs, and their roles in CVDs. Moreover, it also discusses recent advancements in research regarding the diagnostic, therapeutic, prognostic, and monitoring potential of EVs in CVDs, with the aim of providing innovative insights and perspectives on future research directions.

## Overview of extracellular vesicles

**Fig. 1:**
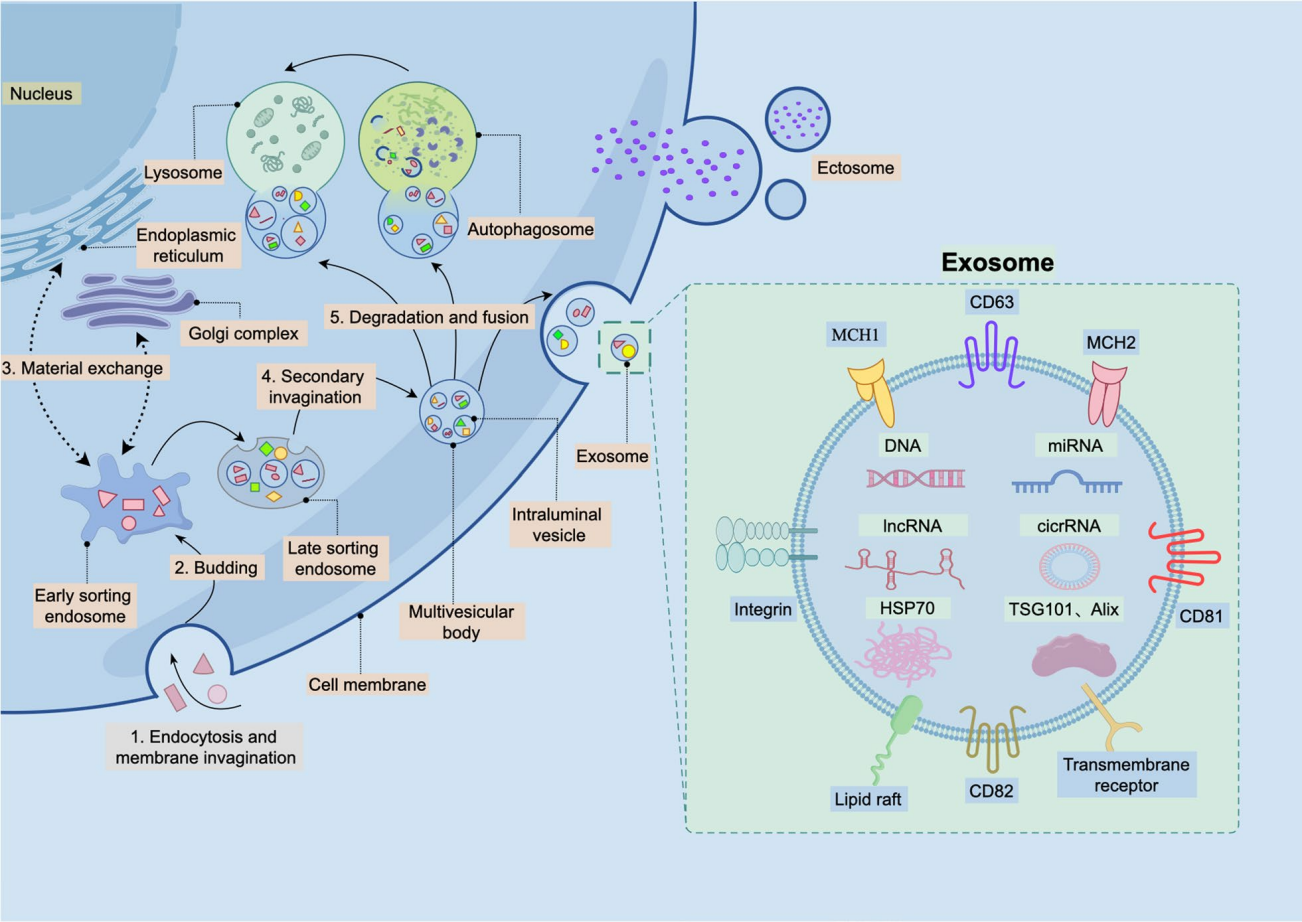
Biosynthetic pathways and molecular structure of exosomes. The biosynthesis of extracellular vesicles is complex multistep process. Ectosomes are released into the extracellular environment through the budding of the plasma membrane. Exosomes involve the coordinated interaction of multiple biosynthetic pathways within the cell. This process encompasses the formation and maturation of endosomes, the loading and exchange of cargo, and the release of exosomes. Exosomes typically contain various components such as cytoskeletal proteins, tetraspanins, molecular chaperones, and a range of proteins involved in biosynthetic processes. Additionally, exosomes transport unique cargo, which encompasses specific nucleic acids, lipids, and proteins, among other substances

EVs are a category of double-layered membrane vesicles that are released by all cells under both physiological and pathological states (van Niel et al. 2018). Generally, based on their biogenesis and size, EVs are roughly divided into two categories (Table 1): exosomes and ectosomes (Kalluri and LeBleu 2020). Ectosomes, which vary in size from 50 to 1000 nm, are characterized by their distinctive mechanism of release that involves outward budding of plasma membrane. As representatives of EVs, exosomes have a diameter ranging from 40 to 160 nm, with an average size of approximately 100 nm. Their precise biosynthesis process remains ambiguous but generally includes the following steps: (Fig. 1): firstly, extracellular components (such as proteins, lipids, nucleic acids, etc.) and cell surface components are internalized into cells through endocytosis and membrane invagination by stress; Afterwards, the cup-shaped structure, composed of the components that enter the cell, comes into being in the form of plasma membrane budding, which undergoes material exchange with the endoplasmic reticulum and -Golgi to develop into early sorting endosomes (ESEs), or merges with pre-existing ESEs; subsequently, ESEs mature into late sorting endosomes (LSEs); finally, the LSEs undergo secondary invagination of the plasma membrane, resulting in the formation of multivesicular body (MVB) that contains multiple intraluminal vesicles (ILVs), which are also referred to as future exosomes. MVBs can fuse with the plasma membrane and release exosomes into the extracellular space through exocytosis. Alternatively, they can be degraded by combining with autophagosomes or directly fusing with lysosomes (Joshi et al. 2020; Kalluri and LeBleu 2020). In addition to the above classification methods, EVs can be further categorized into exosomes (30–150 nm), microvesicles (0.1–1 μm), and apoptotic bodies (1–5 μm) (Boulanger et al. 2017; Gao et al. 2019), or classified as small EVs (50–150 nm), medium EVs (200–800 nm), and Large EVs (≥ 1000 nm) based on their size (Medzhitov et al. 2011). The common components of exosomes encompass cytoskeletal components (actin, myosin, tubulin), tetraspanins (CD9, CD63, CD81, CD82), and molecular chaperones (HSP70, HSP90), along with proteins implicated in exosome biogenesis (Tsg101, Alix) (Pegtel and Gould, 2019). Furthermore, exosomes also contain unique cargoes, such as certain miRNAs, enzymes, and lipids, which can be transported between cells and impact the function of recipient cells (Doyle and Wang 2019; Krylova and Feng 2023). Due to the complexity of their components and their function as messengers, EVs are recognized to play a significant role in intercellular communication (Berumen Sánchez et al. 2021).

**Table 1:** Classification of extracellular vesicles (EVs)

	Classification methods	Category	Size
EV	I	Exosomes	40–160 nm
		Ectosomes	0.1–1 μm
	II	Exosomes	50–150 nm
		Microvesicles	100–1000 nm
		Apoptotic body	1–5 μm
	III	Small EVs	50–150 nm
		Medium EVs	200–800 nm
		Large EVs	≥ 1000 nm

## Exosomes and MI

### Changes in EVs and potential biomarkers in MI

It has been documented that circulating EVs undergo alterations in both their quantity and cargo in response to diverse pathological conditions (Laura Francés et al. 2023; Shah et al. 2018). In animal experiments, an upregulation of miRNA-155 derived from EVs (EVs-miR-155) was identified in both cardiac tissue and plasma of mice 3 days post-MI, in comparison with the control group (Liu et al. 2020b). Additionally, the levels of miRNAs derived from EVs, including miR-1, miR-208, miR-499, and miR-21 in plasma or serum were significantly higher in mice several hours post-acute myocardial infarction (AMI) than sham-operated mice (Cheng et al. 2019; Gu et al. 2018; Yang et al. 2018). In human blood samples, patients who are 6 h post-AMI exhibit increased concentrations of peripheral blood EV-miR-503 in contrast to individuals without AMI (Sun et al. 2022). Furthermore, it was discovered by Naveed Akbar et al. that the quantity of circulating EVs-miRNA-126-3p/-5p were substantially elevated in AMI patients within 48 h compared to the 6-month follow-up of AMI patients (Akbar et al. 2017). Moreover, circulating EVs containing miR-194, miR-34a, and the long non-coding RNA (LncRNA) LIPCAR have been identified as potential biomarkers for predicting cardiac remodeling one year post-MI (Matsumoto et al. 2013; Turkieh et al. 2024). Previous studies have indicated that the risk of major adverse cardiovascular events (MACE) can be predicted by the concentration of circulating EVs in patients after endarterectomy (Timmerman et al., 2022; Verwer et al., 2023). In a retrospective analysis, multivariable Cox regression demonstrated a positive association between circulating levels of EVs-lncRNA-LNC-000226 and the risk of MACE within one year following stent implantation in patients with AMI (Gu et al., 2024). Additionally, studies conducted by Felix Jansen et.al. revealed that during the follow-up period, elevated levels of miR-126 and miR-199a in circulating EVs from patients with stable coronary artery disease (CAD) were significantly correlated with a reduced occurrence of MACE (Jansen et al., 2014). These studies suggest that cargo enclosed in circulating EVs hold potential as biomarkers for MI and could have predictive value for patient outcomes. 

### Adverse role of EVs in MI

Ischemic events have been well-indicated to induce stress, damage, or necrosis in endothelial cells (ECs) and cardiomyocytes (CMs), leading to the release of vesicle-enclosed danger-associated molecular patterns (DAMPs) such as HSP, high mobility group box 1 (HMGB-1), and interleukin-1 (IL-1). These DAMPs interact with pattern recognition receptors of the innate immune system, initiating an inflammatory cascade that includes macrophage activation, leukocyte chemotaxis, and T cell differentiation (Kerr et al. 2018; Medzhitov et al. 2011; Newton and Dixit 2012; Vandervelde et al. 2006). During the onset of AMI, proliferator-activated receptor gamma co-activator 1β and Sirtuin3 were silenced in CMs by ECs-EVs-miR-503 to regulate mitochondrial dysfunction, thereby mediating CMs death and exacerbating myocardial injury (Sun et al. 2022). Following the onset of MI, two distinct phases are typically observed: the inflammatory phase and the cardiac repair phase (Prabhu and Frangogiannis 2016). During MI inflammation, EC-EVs enriched with miR-126-3p/-5p can promote splenic monocytes mobilization by inducing the downregulation of plexin-B2 and the upregulation of integrin β2, potentially facilitating the recruitment of monocytes to the infarcted area of the heart (Akbar et al. 2017). Moreover, the inflammatory response within the infarcted heart can be aggravated, characterized by increased release of inflammatory cytokines when ECs and CMs-EVs are internalized by the infiltrated monocytes (Loyer et al. 2018). Additionally, it has been demonstrated that M1 macrophage-EVs-miRNA-155 not only can depress cardiac repair in MI mice by inhibiting the production of nitric oxide synthase (NOS) in ECs and angiogenesis (Liu et al. 2020b), but also increases the incidence of cardiac rupture by suppressing proliferation of cardiac fibroblast (CF) and intensifying inflammation (Wang et al. 2017). Intriguingly, M2 macrophages-EVs containing circular RNA Ube3a have been identified to amplify myocardial fibrosis in AMI mice by promoting excessive proliferation and phenotype transformation of fibroblasts (Wang et al. 2021b). Therefore, cardiac resident cells engage in intercellular communication via EVs, thereby modulating various signaling pathways and accelerating the progression of MI.

### Protective role of EVs in MI

Shortly after MI, dendritic cells (DCs) migrate to the infarcted region to regulate the immune response (Kretzschmar et al. 2012). Research has demonstrated that the administration of DCs into MI mice can stimulate the activation of regulatory T (Treg) cells and facilitate the conversion of M1 macrophages to M2 macrophages, leading to enhanced wound remodeling and improved survival (Choo et al. 2017). Treg cells that highly express SPARC (secreted protein acidic and cysteine rich) release EVs which can effectively reverse cardiac function dysfunction after acute myocardial infarction (AMI) by suppressing the expression of pro-inflammatory cytokines like IL-1β and promoting collagen synthesis (Cheng et al. 2022; Xia et al. 2020). Furthermore, circulating-EVs can mobilize bone marrow progenitor cells by downregulating CXCR4 in these cells, which may aid in cardiac repair within the infarcted area (Cheng et al. 2019). Moreover, the left ventricular ejection fraction (LVEF) in AMI rats can be also enhanced by cardiac telocytes-EVs-miR-21, which can promote angiogenesis and inhibit apoptosis in ECs (Liao et al. 2021). Additionally, ECs-EVs-Circ Whsc1 has been discovered to encourage CMs proliferation and alleviate cardiac fibrosis in MI mice by activating the TRIM59/STAT3/CyclinB2 pathway (Wei et al. 2023). These studies reveal the crucial role of EVs in the progression and amelioration of MI (Table 2).

**Table 2:** The role of EVs in the progression of myocardial infarction (MI)

Origin	Cargo	Target cell	Model	Target gene/pathway	Function	Refs.
Mice plasma	miR-1a	BMPC	Mice AMI	CXCR4	Progenitor cell mobilization↑	Cheng et al. (2019)
Human serum (healthy)	miR-21	CM	Mice AMI	PDCD4	CM apoptosis↓, infarction area↓	Gu et al. (2018)
CM	miR-21	HUVEC, CM	Mice MI	PDCD4	EC apoptosis↓, cardiac function↑	Song et al. (2019)
CM	miR-208a	CF	Rat MI	Dyrk2	CF proliferation↑, differentiation to myo-CF↑, fibrosis↑, cardiac function↓	Yang et al. (2018)
CM, EC	Unknow	Monocyte	Mice MI	Unknow	Inflammation↑	Yang et al. (2018)
MAEC	miR-503	CM	Mice AMI	PGC-1β, SIRT3	Mitochondrial dysfunction↑, apoptosis↑, cardiac function↓	Sun et al. (2022)
HUVEC	miRNA-126-3p/5p	Monocyte	Mice MI	Plexin-B2/ITGB2	Monocyte mobilization↑	Akbar et al. (2017)
HCMEC	Circ Whsc1	CM	Mice MI	TRIM59/STAT3/CyclinB2	CM proliferation↑, cardiac function↑	Wei et al. (2023)
BMDM	miR-155	CF	Mice MI	Sos1/Socs1	CF proliferation↓, inflammation↑, cardiac function↓	Wang et al. (2017)
BMDM	miR-155	HCAEC	Mice MI	RAC1-PAK2, Sirt1/AMPKα2	EC proliferation↓, angiogenesis↓, cardiac function↓	Liu et al. (2020b)
BMDM	Circ Ube3a	CF	Mice AMI	miR-138-5p/RhoC	Myofibroblast transformation↑, fibrosis↑, cardiac function↓	Wang et al. (2021b)
Treg cell	Unknow	Unknow	Mice MI	Unknow	Inflammation↓, collagen↑, cardiac function↑	Cheng et al. (2022)
Cardiac telocyte	miR-21-5p	EC	Rat MI	Cdip1	EC apoptosis↓, angiogenesis↑, cardiac function↑	Liao et al. (2021)

*BM-PC* Bone marrow progenitor cell, *CM* cardiomyocytes, *HUVEC* human umbilical vein endothelial cell, *CF* cardiac fibroblast, *PM* peritoneal macrophage, *MAEC* mouse arterial endothelial cell, *HCMEC* human cardiac microvascular endothelial cell, *BMDM* bone marrow-derived macrophage, *HCAEC* human coronary endothelial cell, *Treg* regulatory T cell

### Cell-free EV-based therapy for MI

Increasing evidence indicates that miR-21 act as cardioprotective factor in MI (Gu et al. 2018; Wei et al. 2021). Therefore, utilizing EVs from cells highly expressing these miRs may represent a promising strategy for cell-free therapy in MI (Luo et al. 2017; Song et al. 2019). For instance, exosome-like nanocomplexes have been developed to protect miR-21from degradation, and these engineered EVs effectively target to injured CMs, thereby inhibiting programmed cell death 4 (PDCD4) in CMs and enhancing myocardial repair in MI mice (Yao et al. 2020). Recent research has indicated that cardiac-derived cells (CDCs)-EVs contain yREX3, a type of Y RNA, which enhances cardiac repair in MI mice by promoting macrophage efferocytosis (Ciullo et al., 2024). Furthermore, EVs secreted by human induced pluripotent stem cell (hiPSC)-cardiac cells can improve cardiac function in large animal models of MI by modulating CM apoptosis and proliferation (Gao et al. 2020; Jung et al. 2021). In recent years, a growing body of research has revealed that mesenchymal stem cells (MSCs)-EVs of diverse types contain miRNAs, such as miR-486-5p and miR-210, which can mitigate cardiac injury in MI mice by targeting multiple signaling pathways, including enhancing autophagy (Liu et al. 2020a), reducing CM apoptosis (Song et al. 2021), and stimulating angiogenesis (Li et al. 2021e). Additionally, research have indicated that LncRNA H19 also exerts a protective effect in MI by regulating different cell function, such as modulating CM apoptosis, CF activation and inflammation (Hobuß et al. 2020). It was discovered that the proliferation and migration of ECs, angiogenesis, as well as cardiac function can be enhanced by MSCs pre-treated with atorvastatin through the upregulation of LncRNA H19 expression in EVs (Huang et al. 2020). Moreover, an exosome delivery platform has been engineered by Yuan et al., where biocompatible microneedle patches loaded with MSC-EVs containing anti-fibrotic miR-29b mimics were implanted into the injured heart of MI mice, enhancing the retention of EVs in the infarcted myocardium and more efficiently promoting cardiac repair (Yuan et al. 2023). Interestingly, cardiac progenitor cells (CPCs)-EVs are incapable of promoting CM proliferation, but they may improve heart function in infarcted heart by modulating to fibrosis related genes (Lima Correa et al. 2021). In contrast, Carolina Balbi et al. discovered that osteopontin is present in CPCs-EVs and induces CMs proliferation (Balbi et al. 2021). This discrepancy could be attributed to differences in CPC sources or variations in the preprocessing methods employed in the study. Future investigations should elucidate whether CPC-EVs can modulate the cell cycle of CMs and whether they possess the potential to stimulate CM proliferation.

## Exosomes and ischemia–reperfusion (I/R) injury

### Changes in EVs and potential biomarkers in I/R

It has been documented that the increased production of reactive oxygen species (ROS), intracellular calcium overload, and excessive inflammatory responses may collectively contribute to cardiac cell and tissue damage during I/R injury (Eltzschig and Eckle 2011; Yellon and Hausenloy 2007). Hydrogen peroxide (H_2_O_2_), as a ubiquitous ROS within the body, is commonly used in *in vitro *experiments to induce oxidative stress, mimicking the I/R environment (Sies et al. 2017). Research has revealed that mild H_2_O_2_ stimulation of HEK293 cells enhances the release of EVs (Yang et al. 2019), and the elevation of ROS within vascular smooth muscle cells(VSMCs) facilitates the release of calcium-rich EVs (Furmanik et al. 2020). Moreover, increased ROS levels can also activate the NOD-like receptor thermal protein-associated domain-related protein 3 inflammasome, thereby amplifying the release of EVs enriched in IL-1β (Li et al. 2021a). *In vivo,* increasing evidence indicates that levels of EV-miRs, including miR-133b, miR-208b, miR-499, and miR-155-5p, are significantly elevated in the circulation of I/R model compared to control groups (Deddens et al. 2016; Gidlöf et al. 2011; Hu et al. 2023). These results indicate that the quantity and cargo of EVs can be altered by oxidative stress, potentially serving as a real-time monitoring indicator for oxidative stress-related events in I/R injury.

### Adverse role of EVs in I/R

EVs have been substantiated to influence the pathological process of I/R injury through intercellular communication (Table 3). In myocardial I/R injury mice, cardiac tissue-EVs-miR-155-5p has been discovered to facilitate macrophage polarization towards the M1 phenotype by activating the JAK2/STAT1 signaling pathway, which can lead to exacerbating inflammatory response and heart injury (Ge et al. 2021). In another experiment, circulating EVs-miR-155-5p in I/R mice were observed to reduce ubiquitination of cyclophilin D in CMs by specifically targeting the expression of the NEDD4 protein (a component of the E3 ubiquitin ligase family), which can result in enhancing CM apoptosis and infarct size (Hu et al. 2023). However, these findings do not specifically identify the cell origin of the EVs in the research, necessitating further investigation into the sources of these vesicles during myocardial I/R injury.

**Table 3:** The role of EVs in ischemia–reperfusion (I/R)

Origin	Cargo	Target cell	Model	Target gene/pathway	Function	Refs.
CM	LncRNA ROR	CMEC	Rat I/R	miR-145-5p/p70s6k1/eNOS	EC apoptosis↓, NO↑, infarction area↓	Chen et al. (2020)
HUVEC	Proteins	CM	In vitro	Unknow	CM apoptosis↓	Yadid et al. (2020)
Human serum platelet lysate	Proteins and miRs	CM, CMEC	Mice I/R	Unknow	CM apoptosis↓, angiogenesis↑, cardiac function↑	Livkisa et al. (2024)
Plasma (Healthy human)	Unknow	CM	In vitro	ERK1/2	CM apoptosis↓	Li et al. (2019a)
Mice serum	miR-155-5p	CM	Mice I/R	NEDD4	CM apoptosis↑, inflammation↑, infarction area↑, cardiac function↓	Hu et al. (2023)
Injured heart tissue (Mice)	miR-155-5p	BMDM	Mice I/R	JAK2/STAT1	Inflammation↑, cardiac function↓	Ge et al. (2021)
CF	miR-423-3p	CM	Rat I/R	Rap 2C	CM apoptosis↓, infarction size↓	Luo et al. (2019)
PM	miR-148a	CM	Rat I/R	TXNIP	CM pyroptosis↓, infarction area↓, inflammation↓	Dai et al. (2020)

*CMEC* cardiac microvascular endothelial cell, *PM* peritoneal macrophage

### Protective role of EVs in I/R

Research has demonstrated that plasma-EVs of healthy individuals possess the potential to mitigate oxidative stress-induced CM apoptosis (Li et al. 2019a). During the acute phase of I/R injury, CM apoptosis and infarcted size can be mitigated by CFs-EVs-miR-423-3p through downregulating Ras-related protein 2c in CMs (Luo et al. 2019). In a different study, it has been identified by Yadid et al. that ECs-EVs under hypoxic conditions potentially deliver protective proteins to injured CMs, boosting the respiratory capacity of CMs and attenuating the loss of contractile function caused by I/R injury (Yadid et al. 2020). Additionally, M2 macrophages-EVs, enriched with miR-148a, have been illustrated to decrease inflammasome-mediated pyroptosis in CMs and protect the heart from I/R-induced injury (Dai et al. 2020). Therefore, if it is possible to produce these cardioprotective vesicles in large quantities, it may potentially serve as a therapeutic strategy to improve clinical outcomes in I/R injury.

### Cell-free EV-based therapy for I/R

It has been recorded that miR-126 functions as a cardioprotective nucleic acid, preventing myocardial injury induced by tissue ischemia (Chen et al. 2017; Shi et al. 2020). Bher et al. engineered an exosome-like vehicle encapsulating miR-126 for delivery to the ischemic border zone in MI rat, which led to a reduction in infarct size and cardiac hypertrophy (Bheri et al. 2023). It has been revealed by Dora Livkisa et al. that human serum converted platelet lysate can rapidly produce abundant EVs, which are enriched with various trophic factors and cardioprotective miRNAs to improve I/R mice (Livkisa et al. 2024). Moreover, the biomimetic delivery system based on platelet membranes and biomaterial has been asserted to exhibit superior efficacy in targeting the site of cardiac injury and mitigating adverse cardiac remodeling resulting from myocardial I/R injury, as compared to conventional EVs (Jiang et al. 2023; Li et al. 2021d; Pezzana et al. 2022). Recent research has indicated that Tongxinluo, a traditional Chinese medicine, has markedly improved clinical outcomes for patients with STEMI at both 30 days and 1-year post-treatment (Yang et al., 2023).Furthermore, CM-EVs exposed to Tongxinluo encompass LncRNA-regulator of reprogramming that activates endothelial NOS to enhance cell viability, thereby preserving vascular homeostasis in I/R injury rats upon internalization by ECs (Chen et al. 2020). In recent years, a growing body of experimental evidence has validated the therapeutic benefits of MSCs-EVs in the management of myocardial I/R injury (Chang et al. 2023). MSC-EVs enriched cardioprotective molecules such as manganese superoxide dismutase (Yao et al. 2019a), miR125a-5p (Gao et al. 2023), and miR-455-3p (Wang and Shen 2022) have been demonstrated to improve cardiac function, reduce oxidative stress, and alleviate cardiac inflammation in myocardial I/R injury model (Arslan et al. 2013). Thus, these new research findings will provide novel perspectives and directions for exploring potential cell-free therapy strategies for I/R.

## Exosomes and heart failure (HF)

### Changes in EVs and potential biomarkers in HF

Recent progress in the field has indicated circulating EVs-miRs as valuable non-invasive biomarkers for diagnosing and predicting outcomes in HF patients (Gokulnath et al. 2023; Tian et al. 2023; Xue et al. 2020). It has been evidenced that there is a significant increase in immune cells-EVs in plasma of chronic HF patients and the level of these EVs correlates positively with the severity of HF (Vilella-Figuerola et al. 2022). Furthermore, EVs-miR-92b-5p has been observed to elevate in the serum of HF patients compared to non-CVD patients and healthy volunteers and may serve as a biomarker for the diagnosis of HF (Wu et al. 2018; Zhou et al. 2020). It has been discovered that low expression of miR-30d in circulating EVs is associated with adverse cardiac remodeling following HF (Li et al., 2021b). Moreover, serum-miR-30d levels were significantly reduced in acute HF patients who did not survive a one-year follow-up period compared to those who did (Xiao et al., 2017). Consequently, miR-30d may serve as a prognostic biomarker for clinical outcomes in HF patients.

### Adverse role of EVs in HF

Cardiac insufficiency can be triggered by multiple factors, including hypertension and ventricular hypertrophy, among others, leading to reduced cardiac output (Tanai and Frantz 2015). Hannah Key et al. discovered that circulating-EC-EVs from hypertensive individuals may potentially increase the risk of HF by upregulating the expression of proteins associated with cardiac hypertrophy and fibrosis, while concurrently reducing the expression of NOS (Hannah K Fandl et al., 2023). In HF animal models, CM hypertrophy and left ventricular remodeling can be induced by CFs-EVs enriched with miR-21-3p (Bang et al. 2014), miR-146a (Oh et al. 2018), and miR-27a (Tian et al. 2018), which regulate various signaling pathways in CMs. Furthermore, EVs-miR-494-3p from CMs under pressure overload conditions has been indicated to promote cardiac fibrosis in mice by inhibitingPTEN and promoting the phosphorylation of AKT in CFs (Tang et al. 2023a). An in vitro investigation has demonstrated that CF-EVs can induce CMs hypertrophy by activating the PI3K/Akt and MAPK pathways, which may contribute to HF through the activation of renin angiotensin system (Lyu et al. 2015). CMs and CFs regulate the progression of HF through EVs-mediated intercellular communication, which may represent a potential therapeutic strategy for improving cardiac function in the future.

### Protective role of EVs in HF

It has been identified that ventricular remodeling and cardiac function can be improved in HF animals by CMs-EVs-miRs, including miR-378and miR-30d (Li et al. 2021b; Yuan et al. 2018), which inhibited CM apoptosis and collagen synthesis by modulating MAPK phosphorylation and fibrosis-related gene expression in cardiac cells. Intriguingly, myocardial hypertrophy and fibrosis in cardiac dysfunction mice can also be ameliorated by EVs from ECs treated with specific manipulations, such as CD151 knockdown. Proteins from these EVs can reduce CM hypertrophy and fibroblast proliferation by activating the Peroxisome proliferator-activated receptors signaling pathway (Jiang et al. 2024a). EVs have been extensively examined in HF (Table 4), not only revealing their significant roles in the pathological processes of HF but also providing new perspectives and potential therapeutic targets for HF.

**Table 4:** The role of EVs in heart failure (HF)

Origin	Cargo	Target cell	Model	Target gene/pathway	Function	Refs.
CF	miR-21-3p	CM	Rat TAC	SORBS2, PDLIM5	CM hypertrophy↑, cardiac function↓	Bang et al. (2014)
CF	RNAs, DNAs, proteins	CM	In vitro	PI3K/Akt, MAPK	CM hypertrophy↑	Lyu et al. (2015)
CF	miR-146a	CM	Mice TAC	SUMO1	CM contractility↓, cardiac function↓	Oh et al. (2018)
CF	miR-27a, miR-28a, miR-34a	CM	Mice LCAL	Nrf2/ARE	CM hypertrophy↑, cardiac function↓	Tian et al. (2018)
CM	miR-378	CF	Mice TAC	MKK6/p38 MAPK	CF proliferation↓, fibrosis↓, cardiac function↑	Yuan et al. (2018)
CM	miR-494-3p	CF	Mice TAC	PTEN	CF proliferation↑, fibrosis↑, cardiac function↓	Tang et al. (2023a)
CM	miR-30d	CM, CF	MI Rat, mice I/R	MAP4K4, ITGA5	CM apoptosis↓, fibrosis↓, cardiac function↑	Li et al. (2021b)
HUVEC	Proteins	CF, CM	Mice TAC	PPAR	CM hypertrophy↓, CF proliferation↓, cardiac function↑	Jiang et al. (2024a)

*TAC* transverse aortic constriction, *LCAL* left coronary artery ligation

### Cell-free EV-based therapy for HF

Reportedly, EVs derived from MSCs hold therapeutic potential for improving HF. For instance, MSC-EVs-miR-1246 have been documented to promote neovascularization in chronic HF mice by inhibiting serine proteinase23 and blocking Snail/α-α-SMA signaling in ECs (Wang et al. 2021c). Additionally, El Harane et al. discovered that hiPSC-derived CPCs-EVs, enriched with cardioprotective miRNAs, may stimulate transcriptomic alterations, promote the survival of stressed CMs, and increase LVEF when they are delivered to the infarcted heart tissue of chronic HF mice (El Harane et al. 2018). Future research should further identify the therapeutic mechanisms and biomolecular constituents by which these EVs mediate their therapeutic effects in HF, moving them closer to the clinic.

## Exosomes and atherosclerosis (AS)

### Changes in EVs and potential biomarkers in AS

Emerging evidence indicates that circulating EVs may act as direct indicators for assessing the progression of AS in patients (Della Corte et al. 2023; Wang et al. 2021a) (Table 5). Research has shown that individuals with hypertension, obesity, diabetes, and dyslipidemia—conditions recognized as predisposing factors for AS—display notable changes in EVs in their plasma (Freeman et al. 2018; Martinez-Arroyo et al. 2021; Thomou et al. 2017; Wang et al. 2020b; Yu et al. 2023). Several research has observed increased concentrations of EVs-RNAs including miR-23a-3p and Circ-0001785 (Peng et al. 2022; Tong et al. 2023), within AS plaques and elevated levels of circulating EV-miRs in AS patients or mice compared to non-atherosclerotic groups, such as miR-126-5p, miR-212-3p, and miR-92a-3p (Choi et al. 2023; Liu et al. 2019). Additionally, it has been reported that circulating EVs-miR-133a abundance can predict AS plaque progression and cardiovascular events in familial hypercholesterolemia patients (Escate et al. 2021), as well as the quantities of circulating EVs-Circ RNA CDR1as are positively associated with inflammation in AS (Yang et al. 2024). Thus, EVs are emerging as promising biomarkers for the prediction and diagnosis of AS.

**Table 5:** The role of EVs in atherosclerosis (AS)

Origin	Cargo	Target cell	Model	Target gene/pathway	Function	Refs.
Monocytes	Circ-0001785	HUVEC	Mice AS	miR-513a-5p	EC apoptosis↓, proliferation↑, lesion area↓, cardiac function↑	Tong et al. (2023)
Plasma	miR-182-5p	HUVEC	Mice AS	MYD88/NF-ĸB/NLRP3	EC injury↓, inflammation↓, lesion area↓	Li et al. (2023b)
Serum	miR-186-5p	Macrophage	Mice AS	LOX-1	Foam cell↓, lipid deposition↓, lesion area↓	Ding et al. (2022)
Plaque	miR-23a-3p	EC	Mice AS	Dusp5	Inflammation↑, monocyte recruitment↑	Peng et al. (2022)
HPB	Rap1	HASMC	Mice AS	ERK/p38 MAPK	Inflammation↑, SMC migration and proliferation↑	Perdomo et al. (2020)
HCAEC	miR-92a-3p	HCAEC	In vitro	THBS1	EC proliferation↑, migration↑	Liu et al. (2019)
HUVEC	Circ-0086296	HUVEC	Mice AS	miR-576-3p	Angiogenesis↓, ECs injury↑, inflammation↑, lesion area↑	Zhang et al. (2022a)
HUVEC	Unknow	Macrophage	In vitro	MAPK/NF-kB	M1ɸ polarization↑, lipid deposition↑, inflammation↑	Lin et al. (2023)
HUVEC	LINC01005	HASMC	In vitro	miR-128-3p/KLF4	VSMC phenotype switch↑, proliferation↑, migration↑	Zhang et al. (2020b)
HUVEC	miR-126-5p, miR-212-3p	Monocyte	Mice AS	Unknow	Inflammation↑, foam cell↑, lesion area↑	Choi et al. (2023)
Macrophage	miR‐4532	HUVEC	In vitro	SP1	ICAM‐1↑, VCAM‐1↑, endothelial dysfunction↑	Choi et al. (2023)
BMDM	miR-21-3p	VSMC	Mice AS	PTEN	VSMC proliferation↑, migration↑, lesion area↑	Zhu et al. (2019)
BMDM	miR-486–5p	Macrophage	Mice AS	Abca1	M1ɸ proliferation↑, lesion area↑	Bouchareychas et al. (2021)
BMDM	miR-99a, miR-146b, miR-378a	Macrophage	Mice AS	NF-κB/TNF-α	M2ɸ polarization↑, lesion area↓	Bouchareychas et al. (2020)
HCASMC	miR-221, miR-222	HUVEC, HCAEC, monocyte	Mice AS	QKI	ICAM-1↑, inflammation↑, lesion area↑	Yu et al. (2023)
HASMC	miR-155	HCMEC	Mice AS	Unknow	Endothelial injury↑, lesion area↑	Zheng et al. (2017)
Platelet	miR-25-3p	CVEC	Mice AS	Adam10	Inflammation↓, lipid deposition↓, lesion area↓	Yao et al. (2019b)
Platelet	miR-223	HUVEC	Mice AS	NF-κB/MAPK	ICAM-1↓, inflammation↓, thrombosis↓	Li et al. (2017)
Visceral adipocyte	miR-27b-3p	HUVEC	Mice AS	PPARα	Inflammation↓, plaque formation, lesion area↑	Tang et al. (2023b)

*HPB* human peripheral blood, *HASMC* human aortic smooth muscle cell, *VSMC* vascular smooth muscle cell, *CVEC* coronary vascular endothelial cell

### Adverse role of EVs in AS

It has been demonstrated that circulating EVs rich in Rap1 protein or miR-27b-3p can increase the risk of AS in individuals with metabolic syndrome or obesity by activating inflammatory signaling pathways such as ERK5/P38 and NF-κB (Perdomo et al. 2020; Tang et al. 2023b). Additionally, recent research has revealed that under the stimulation of oxidized low-density lipoprotein (ox-LDL), nicotine, and high glucose, miRs such as miR-4532, miR-21-3p, and miR-486-5p within macrophages-EVs may exacerbate endothelial dysfunction and promote AS progression by upregulating VCAM‐1 in ECs (Liu et al. 2022), enhancing the proliferation and migration of VSMCs (Zhu et al. 2019), and promoting macrophage proliferation(Bouchareychas et al. 2021). Besides, upon stimulation with ox-LDL, RNA molecules like Circ-0086296 and LINC01005 present in ECs-EVs can facilitate the phenotypic switch and proliferation of SMCs, aggravate EC injury and induce inflammation via intercellular communication, consequently expediting the development of AS (Zhang et al. 2022a; Zhang et al. 2020b). Subsequently, under the influence of inflammation or other external stimuli, ECs-EVs can also transmit information, such as miR-126-5p and miR-212-3p, to monocytes. This process intensifies AS by promoting the the differentiation of monocytes, the formation of foam cells, and the exacerbation of inflammation (Choi et al. 2023; Lin et al. 2023). Furthermore, it has been substantiated that plaques formation can be promoted in mice model by VSMCs-EVs, which can upregulate ICAM-1 expression in ECs, mediate endothelial injury and induce monocytes to polarize towards pro-inflammatory phenotype (Yu et al. 2023; Zheng et al. 2017). Future research should further elucidate and define the signaling pathways targeted by these EVs, which can be thoroughly analyzed through techniques such as high-throughput sequencing and single-cell RNA sequencing.

### Protective role of EVs in AS

It has been documented that a decrease in circulating EV-miRs, including miR-182-5p and miR-186-5p, in patients with AMI or insomnia may accelerate AS progression by enhancing the macrophage uptake of ox-LDL, inducing endothelial dysfunction, and promoting inflammation, which implicates an indirect cardioprotective role of EVs in AS (Ding et al. 2022; Li et al. 2023b). Likewise, heme within erythrocytes-EVs can effectively reduce the accumulation of ox-LDL in macrophages and the number of foam cells, thereby mitigating atherosclerotic lesions in mice (Pham et al. 2023). Furthermore, platelet-EVs-miRs, such as miR-223 and miR-25-3p, have been identified to ameliorate AS lesions in mice by targeting inflammatory pathways such as NF-κB in ECs to reduce the release of inflammatory cytokines (Li et al. 2017; Yao et al. 2019b). Moreover, EC apoptosis can be inhibited and M2 macrophage polarization can be induced by immune cells through EVs-mediated intercellular communication, thereby stabilizing atherosclerotic plaques and reducing lesion burden in mice (Bouchareychas et al. 2020; Tong et al. 2023). Intercellular communication mediated by EVs derived from circulating blood cells and cardiac resident cells can alleviate atherosclerotic inflammatory responses by carrying cardioprotective factors within the vesicles.

### Cell-free EV-based therapy for AS

It has been reported that the expression level of junction adhesion molecule A (JMA-A) is upregulated in AS patients and promotes plaque formation (Schmitt et al. 2014; Zhang et al. 2016). Intriguingly, human umbilical cord MSC(HUCMSC)-EVs-miR-145 was observed to inhibit EC migration in vitro and reduces plaque information in vivo by downregulating JMA-A (Yang et al. 2021). Furthermore, the proliferation and tube formation of ECs can be promoted by endothelial progenitor cells-EVs-miR-21-5p by targeting signal-induced proliferation-related 1-like 2 gene, thereby providing protection against vascular damage caused by AS (Ke et al. 2022). Moreover, strategies for fluorescent labeling of EVs (Ji et al. 2024), EV-based drug delivery platforms (Bu et al. 2021; Zhang et al. 2023b), nanovesicle biomimetic systems (Jiang et al. 2024b), and emerging biotechnologies have been developed to monitor the progression of AS in real-time and deliver drugs more precisely to the plaque area, effectively improving AS conditions. Hence, these studies not only reveal the crucial role of EVs in AS but also lay an important theoretical foundation and practical guidance for the development of novel therapeutic strategies in the future.

## Exosomes and myocarditis

### Changes in EVs and potential biomarkers in myocarditis

Viral infection is recognized as a predominant infectious cause of myocarditis, and increasing evidence points to the presence of viral particles, genomes, or other pathogenic factors within EVs released by virus-infected cells, facilitating their dissemination to neighboring cells (Ammirati and Moslehi 2023; Feng 2020). Furthermore, significant alterations in serum EV levels have been identified in viral myocarditis(VM) patients and mice when compared to controls, including elevated levels of miR-30a, miR-181d (Fan et al. 2019), miR-142 (Sun et al. 2020a), retinol-binding protein 4 (Zhao et al. 2019), and hsa-miR-548a-3p, whereas hsa-miR-500b-5p exhibits a decreasing trend (Wu et al. 2024). Moreover, in contrast to healthy controls, the concentrations of EV-miRs such as hsa-miR-146a-5p, hsa-miR-23a-3p, and hsa-miR-192 have been observed to be significantly increased in the serum of fulminant myocarditis (FM) patients, while hsa-miR-155 and hsa-miR-320a have been demonstrated to show high accuracy in distinguishing FM from non-FM (Zhang et al. 2023d; Zhang et al. 2021).

### The role of EVs of EVs in myocarditis

It has been revealed that over-activation of pyruvate kinase M2 (PKM2) in macrophages can promote the polarization of M1 macrophages, while the expression of pro-inflammatory genes and the glycolytic activity of macrophages are both inhibited in a mouse model with specific deletion of the PKM2 gene in myeloid cells (Doddapattar et al. 2022; Gai et al. 2023; Hou et al. 2020). Contemporary research has indicated that cardioprotective EVs, such as M2 macrophages-EVs, contain LncRNA AK083884 that regulate the PKM2/HIF-α and SOCS2/STAT6 pathways, which inhibits the glycolysis and promotes the polarization of M2 macrophages, thereby reducing inflammation and improving cardiac ejection fraction in VM mice (Zhang et al. 2024b).

By contrast, cardiac dysfunction can be exacerbated in autoimmune myocarditis mice by circulating EVs-miR-142, which can induce metabolic reprogramming of T cells and promote the release of inflammatory cytokines by targeting the expression of PKM2 and inhibiting SOCS1/STAT in CD4+ T cells (Sun et al. 2020a). Additionally, EVs-miR-320 in VM mice serum has been determined to target the phosphoinositide-3-kinase regulatory subunit 1, thereby suppressing the protein kinase B/mammalian target of rapamycin (AKT/mTOR) pathway crucial for cell growth and promoting CM apoptosis (Zhang et al. 2023c). These studies reveal the dual role of EVs in the pathogenesis of myocarditis, where their impact on cardiac function is contingent upon the specific cargo they transport (Table 6).

**Table 6:** The role of EVs in myocarditis

Origin	Cargo	Target cell	Model	Target gene/pathway	Function	Refs.
Serum	miR-30a, miR-181d	CM	Mice VM	SOCS3	Inflammation↑	Fan et al. (2019)
Serum	miR-320	CM	Mice VM	Pik3r1	CM apoptosis↑	Zhang et al. (2023c)
Serum	miR-142	CD4 + T cell	Mice AM	MBD2, SOCS1	Glucometabolic↑, inflammation↑, cardiac function↓	Sun et al. (2020a)
BMDM	LncRNA AK083884	BMDM	Mice VM	PKM2/HIF-1α	M2ɸ polarization↑, inflammation↓, cardiac function↑	Zhang et al. (2024b)

*VM* vital myocarditis, *AM* autoimmune myocarditis, *BMDM* bone marrow-derived macrophage

### Cell-free EV-based therapy for myocarditis

It has been reported that VM can be alleviated through the administration of MSCs-EVs, which can restrain CM apoptosis by activating the AMPK/mTOR pathway and reduce myocardial inflammation via suppressing mastermind-like 1 in CMs (Gu et al. 2020; Li et al. 2021c). Analogously, CPCs-EVs inhibit cell apoptosis by activating Bcl2 expression and inhibiting Bim and Bax. Injection of CPCs-EVs reverses CK-MB and cTnT elevation in coxsackievirus3-induced myocarditis rats, indicating its potential as a treatment for VM (Li et al. 2019b). Remarkably, EVs-based coxsackievirus3 vaccine VP1 has been observed to induce stronger specific T cell proliferation and cytotoxic T lymphocyte responses compared to recombinant VP1 protein vaccines. This results in higher levels of CVB3-specific serum IgG antibodies, effectively clearing the virus and enhancing resistance to CVB3-induced myocarditis in mice (Zhang et al. 2023a). However, this vaccine is still some distance away from clinical translation and future research needs to further investigate its efficacy, toxic side effects and metabolic distribution in humans to ensure the safety of clinical application.

## Exosomes and cardiomyopathy

### Changes in EVs and potential biomarkers in cardiomyopathy

Despite the lack of a unified definition for cardiomyopathy, the diverse factors known to cause it, including the toxicity of chemotherapy drugs, and clinical conditions such as, diabetes, obesity, and bacterial myocarditis, are well recognized (Ciarambino et al. 2021). It has been reported that the levels of EVs-miR-126 in serum significantly increase in sepsis-induced cardiomyopathy mice (Zhang et al. 2020a). Additionally, the expression of miRNA-218-5p is upregulated in CMs-EVs in patients with familial dilated cardiomyopathy (DCM) (Fu et al. 2023). Furthermore, in obesity-related cardiomyopathy mice, the concentrations of miR-122 in circulating EVs are positively correlated with cardiac dysfunction (Wang et al. 2019a). Notably, studies have demonstrated that iPSC-CM-EVs of patients with hypertrophic cardiomyopathy (HCM) mutations that show a notable elevation in the levels of small nucleolar RNAs (snoRNA), such as SNORD 43 and SNORD 166, in transcriptomic analyses when compared to control group (James et al., 2021). An increasing body of research suggests that EVs participate in and influence the progression of cardiomyopathy (Table 7) and their components hold promise as potential biomarkers for cardiomyopathy.

**Table 7:** The role of EVs in cardiopathy

Origin	Cargo	Target cell	Model	Target gene/pathway	Function	Refs.
Serum	miR-126	HUVEC	Mice sepsis	Unknow	ICAM-1↓, VCAM-1↓, inflammation, cardiac function↑	Zhang et al. (2020a)
CM	miR-218-5p	CF	Mice left thoracotomy	TNFAIP3	Fibrosis↑, cardiac function↓	(Fu et al. 2023)
HUVEC	miR-146a	CM	Mice PPCM	ErbB4, Notch1, IRAK1	CM metabolism↓, cardiac function↓	Halkein et al. (2013)
CMEC	Mst1	CM	Mice diabetes	Daxx	Autophagy↓, CM apoptosis↑, CM glucometabolic↓, cardiac function↓	Hu et al. (2018)
Serum	Unknow	CM, macrophage	DOXO-Rat	Unknow	CM apoptosis↓, M1 ɸ polarization↑, inflammation↑	Yarana et al. (2022)
Plasma, liver	miR-122	CM	Mice obesity	Arl-2	CM mitochondrial function↓, cardiac function↓	(Wang et al. 2019a)
BMDM	miR-155	CM	Mice uremia	FoxO3a	CM pyroptosis↑, hypertrophy↑, cardiac function↓	Wang et al. (2020a)

*DOXO* doxorubicin

### The role of EVs in cardiomyopathy

Mammalian sterile 20-like kinases 1, a pivotal regulator of cell growth through the Hippo signaling pathway, has been identified as a prominent component in ECs-EVs of diabetic mice. Not only can the EVs inhibit CMs autophagy and promote cell apoptosis, but also inhibit glycolysis in CMs, thereby worsening diabetic cardiomyopathy (Hu et al. 2018). In uremic cardiomyopathy mice, CM pyroptosis and hypertrophy can be exacerbated by macrophage-EVs- miR-155, which can promote the progression of cardiomyopathy through inhibiting transcription factors FoxO3 expression (Wang et al. 2020a). Furthermore, miR-218-5p in CMs-EVs from DCM patients can activate the TGF-β signaling and increase fibrosis-related gene expression by inhibiting the master inflammation inhibitor TNFAIP3 in CFs, which results in expanded fibrosis and impaired cardiac function when these EVs are injected into mice hearts (Fu et al. 2023). In doxorubicin (DOX)-induced cardiomyopathy animal models, serum EVs protect CMs from oxidative stress damage by upregulating antioxidant enzymes like glutathione peroxidase1. Interestingly, these EVs also activate macrophages to produce ROS and increase levels of IL-6 and TNF-α, intensifying cardiac inflammation (Yarana et al. 2022). The dual role of serum-EVs in the progression of DCM is elucidated in this research. Methods to enhance the cardioprotective effects of these EVs in DCM, specifically by reducing ROS-induced CM apoptosis and macrophage-mediated immune response, need to be further explored. Subsequent research should delve into the cell origins and specific components of these EVs, aiming to harness their potential benefits in the treatment of cardiomyopathy.

### Cell-free EV-based therapy for cardiomyopathy

Emerging research has highlighted the potential of diverse MSC-EVs as a viable cell-free therapeutic strategy for diabetic cardiomyopathy. It has been demonstrated that the condition can be mitigated by these EVs through the inhibition of excessive autophagy in CMs, the facilitation of M2 macrophage polarization, the reduction of apoptosis in CFs and collagen synthesis via intricate signaling pathways (Banerjee and Singla 2024; Zhang et al. 2022b; Zhen et al. 2024). Furthermore, stem cells-EVs are enriched with various cardioprotective RNAs, including miR-199a-3p, miR-9-5p, LncRNA NEAT1, and miR-let-7i, which have been demonstrated to suppress apoptosis, mitochondrial fragmentation, and senescence in CM by enhancing phosphorylation of the Akt pathway (Lee et al. 2021), inhibiting the vascular peroxidase 1/extracellular regulated protein kinase pathway (Zheng et al. 2024), activating Sirt2 (Zhuang et al. 2020), and downregulating the Yes-associated protein 1/Hippo pathway in CMs (Ni et al. 2020), thereby alleviating DOX-induced cardiomyopathy, characterized by reduced inflammation and improved LVEF. Additionally, it has been identified that intravenous administration of CDCs-EVs, containing YRNA-YF-1 or cardioprotective miRNAs, in mice with arrhythmogenic cardiomyopathy and hypertrophic cardiomyopathy resulted in alleviation of cardiac hypertrophy, fibrosis, and cardiac inflammation by reducing CM death and neutrophils mobilization (Huang et al. 2021; Lin et al. 2021). Of note, it has been indicated by Hirai et al. that CDC-EVs-miR-146a-5p can attenuate cardiac fibrosis and enhance cardiac function by facilitating neovascularization and promoting CM proliferation. And their prospective clinical study involving pediatric DCM patients revealed that the infusion of the EVs via coronary artery delivery exhibited favorable safety profiles throughout the one-year follow-up (Hirai et al. 2020). Consequently, EV-based cell-free therapy has shown the promise in improving clinical symptom in cardiomyopathy, potentially emerging as a novel treatment strategy for cardiomyopathy.

## Exosomes and aneurysm

### Changes in EVs and potential biomarkers in aneurysm

It has been reported that the levels of EVs-miR-29a-3p and EVs-miR-145-5p in the plasma of intracranial aneurysms patients are significantly higher than those in healthy individuals (Liao et al. 2020). Similarly, the concentrations of EVs-let-7i-3p in the serum of coronary artery aneurysms patients also exhibit an upward trend (Wang et al. 2019b). Additionally, it has been revealed that within 1 month following endovascular repair of abdominal aortic aneurysm (EVAR), patients with endoleaks exhibit higher concentrations of EC-EVs compared to those without endoleaks, while the levels of platelet-EVs in patients with endoleaks significantly decrease within 6 months post-EVAR (Serafini et al. 2022). These findings suggest that these EVs may serve as non-invasive and specific biomarkers for early detection of EVAR. Interestingly, Lopez et al. detected a significant decrease in circulating EVs-miR-122-5p levels in abdominal aortic aneurysm (AAA) patients compared to those without AAA (Lopez et al. 2023), while Alex Hildebrandt observed that AAA patients with AS had higher circulating EVs-miR-122-5p levels than healthy controls (Hildebrandt et al. 2021). Accordingly, it is imperative conduct further research to elucidate the role of EVs-miR-122-5p in aneurysms, and larger-scale studies are required to clarify the driving mechanisms behind the quantitative and qualitative changes in aneurysm patients.

### The role of EV in aneurysm

An in vitro experiment indicates that M1 macrophages-EVs-LncRNA PVT1 may promote the progression of AAA by inducing inflammation and pyroptosis through the inhibition of HMGB1 in VSMCs (Zhang et al. 2024a). Furthermore, in human thoracic aortic aneurysms, it has been revealed that telocyte-EVs-cargo, such as KLF-4 and miR-146a, can regulate the dedifferentiation of VSMCs into a synthetic phenotype, potentially increasing ECM degradation, MMP production, and susceptibility to aneurysm formation (Aschacher et al. 2022). In in vivo studies, it was observed that T lymphocytes-EVs are enriched with phospholipids containing polyunsaturated fatty acid, which facilitates increased iron accumulation and enhances lipid peroxidation in macrophages, thereby contributing to the advancement of AAA in mice (Dang et al. 2022). Given the relatively limited application of EVs in the study of aneurysms compared to other CVDs (Table 8), it remains uncertain whether cardiac resident cells secrete cardioprotective EVs during the aneurysm formation phase. Future investigations are essential to further explore the multifaceted role of EVs in the progression of aneurysms, aiming to identify more effective therapeutic targets.

**Table 8:** The role of EVs in aneurysm. TAA, thoracic aortic aneurysm

Origin	Cargo	Target cell	Model	Target gene/pathway	Function	Refs.
Telocyte	miRs, proteins	SMC	Patient TAA	Unknow	Synthetic SMC↑, SMC proliferation↑	Aschacher et al. (2022)
T lymphocyte	phospholipids containing PUFA	PM	Mice AAA	Unknow	Macrophage iron accumulation↑, lipid oxidation↑, inflammation↑	Dang et al. (2022)
Macrophage	LncRNA PVT1	VSMC	In vitro	miR-186-5p	VSMC pyroptosis↑, inflammation↑	Zhang et al. (2024a)

*TAA* thoracic aortic aneurysm, *AAA* abdominal aortic aneurysm, *PUFA* polyunsaturated fatty acid

### Cell-free EV-based therapy for aneurysm

EVs derived from various MSCs have been reported to exhibit potential for improving and treating aneurysms under in vitro culture conditions. For instance, the formation of AAA may be inhibited by EVs-miR-19b-3p and miR-17-5p from adipose stem cells(ADSCs), which can suppress mitochondrial fission-related pathway and inflammasome pathway in VSMCs (Hu et al. 2022a; Zhang et al. 2023e). Additionally, several research has indicated that bone marrow derived MSC-EVs not only shift aortic lesion development towards an anti-proteolytic environment by downregulating MMP2 and upregulating tissue inhibitor of metalloproteinase 2 expression in SMCs, but also regulate immune cells such as inhibiting mast cell activation and maintaining Th17/Treg balance by increasing prostaglandin E2 expression and inhibiting the PI3K/Akt/NF-κB signaling pathway, thereby preventing the information of aneurysm and reducing the risk of aneurysm rupture in mice (Liu et al. 2016; Sajeesh et al. 2020; Sun et al. 2020b). Furthermore, it has been discovered by Michael Spinosa et al. that human umbilical cord MSC(HUCMSC)-EVs-miR-147 can mitigate the formation of AAA in mice by inhibiting macrophage activity and reducing elastin degradation(Spinosa et al. 2018). Notably, neutrophil extracellular traps (NETs), a defense mechanism wherein the host utilizes extracellular networks containing bactericidal proteins to capture and eliminate pathogens, have been demonstrated to contribute to the formation of AAA (Meher et al. 2018). Interestingly, HUCMSC-EVs can prevent AAA formation by converting NETosis into neutrophil apoptosis to inhibit NET formation (Chen et al. 2023). However, the study did not pinpoint specific substances within EVs that exhibit anti-NETosis activity. Therefore, future research should further explore the specific cargoes in these EVs to lay the foundation for the quantitative production of therapeutic exosomes for aneurysm treatment.

## Challenges and prospects

Since EVs can be secreted by virtually all cells, they are abundantly present in bodily fluids such as blood, saliva, and urine. The bilayer membrane structure of EVs allows them to store their contents within the human body for extended periods. Recent research has indicated that using exosome-specific membrane proteins as biomarkers for detecting colorectal cancer yields significantly higher areas under the receiver operating characteristic curve compared to traditional carcinoembryonic antigen markers (Li et al. 2023a). Thus, EVs hold promise as non-invasive biomarkers for aiding in disease diagnosis. However, the high heterogeneity of EVs, coupled with the limitations of traditional extraction methods (e.g., differential centrifugation, density gradient centrifugation, size-exclusion chromatography, ultrafiltration, and immunoprecipitation), which are time-consuming, yield low purity, and are costly (Szatanek et al. 2015), often result in contaminated EVs. For instance, the intense centrifugal forces used in ultracentrifugation can compromise the integrity of exosomes, while polymer precipitation techniques may inadvertently co-precipitate some proteins, including lipoproteins, as well as other non-EVs like nucleic acids. Furthermore, during ultrafiltration, exosomes can obstruct the filtration pores, which may compromise the accuracy of experimental results (Kanchi Ravi et al., 2015; Peterson et al., 2015; Soares Martins et al., 2018; Tan et al., 2020). Hence, future research should focus on developing new technologies for the efficient extraction and purification of EVs from body fluids, and on deepening the study of different EV subtypes to identify potential specific markers, which will facilitate the creation of innovative tools and methods for the precise identification and characterization of various types of EVs. In CVD research, a significant number of clinical samples originate from patients who have already received pharmacological treatment, particularly those with diabetes, AS, and hypertension. Nevertheless, medications such as acarbose, insulin analogs, statins, and antihypertensive drugs may alter the quantity and quality of circulating exosomes (Hildebrandt et al. 2021; Jansen et al. 2017; Lopez et al. 2023), thereby influencing study outcomes. Consequently, during the experimental design phase, it is crucial to fully consider the medication status of the patients, minimize the impact of drugs on EVs, and clearly define the inclusion criteria for both the experimental and control groups to ensure the accuracy of the research results. The MISEV2023 guidelines provide a series of recommendations for the collection, preprocessing, separation, and concentration of EVs. For instance, it is advised that the characteristics of the EV source be provided prior to the commencement of the study, that the providers undergo overnight fasting before sampling, that storage parameters before and after EV separation be reported, and that the properties of the materials used during the concentration process be considered, among other measures. These actions contribute to enhancing the extraction purity in EV research (Welsh et al., 2024). In addition, in patients with AS, aneurysms, and obesity-related cardiomyopathy, the levels of circulating exosomes-miR-122 have been observed to change (Liu et al. 2020c; Lopez et al. 2023; Wang et al. 2019a). Similarly, elevated concentrations of circulating exosomes-miR-21 have been reported in cases of HF, most solid tumors, and preeclampsia (Hu et al. 2022b; Lu et al. 2020; Thum et al. 2008). These findings suggest that there may be similarities in EV alterations across different cardiovascular and systemic diseases and not all EV changes are disease specific. Consequently, relying solely on a single circulating EV as a diagnostic and prognostic biomarker for CVDs may be limited. The integration of multiple EV biomarkers could potentially enhance their clinical utility. Future investigations may require extensive prospective studies utilizing population baseline data and circulating miRNA profiles across various CVDs to validate the efficacy of different combinations of circulating miRNAs as CVD biomarkers.

It has been documented that stem cell therapy was widely applied in clinical treatment and several stem cell treatment drugs were approved for market release globally, such as Cartistem (Park et al. 2017), Alofisel (Verstockt et al. 2018), and Prochymal (Murata and Teshima 2021). Yuto Nakamura et al. discovered that the injection of MSCs could improve cardiac function in HF mice, with this effect primarily attributed to the EVs secreted by these MSCs (Nakamura et al. 2020). Given the highly similar growth characteristics of stem cells to tumor cells, uncontrolled differentiation and proliferation processes may lead to tumorigenesis (Knoepfler 2009). By contrast, EVs-based therapies exhibit significant advantages, including low tumorigenic risk, stable long-term preservation, diverse delivery methods, and virtually no ethical concerns (Ma et al. 2023). Nonetheless, the limited yield and off-target effects of natural EVs hinder the practical application of EVs-based cell-free therapies in clinical settings. The production of EVs can be effectively enhanced through methods such as selecting parent cells with high activity and large yield, pretreating parent cells, employing three-dimensional cell culture systems, drug loading, and genetic modification (de Abreu et al. 2020; Han et al. 2023). Additionally, chemical engineering of exosomes, surface modification, and nanotechnology hold significant promise in targeted drug delivery (Koh et al. 2023). MSCs and iPSCs are two common types of stem cells, and their derived EVs have been widely utilized in research aimed at disease improvement (Chi et al., 2024; Cone et al., 2021; Levy et al., 2023). However, the differences in therapeutic efficacy between these two types of stem cell-EVs in certain diseases remain unclear (Tan et al., 2024). Future studies could explore the therapeutic efficacy of EVs derived from different types of stem cells in specific diseases, aiming to identify the most appropriate cells for optimizing effective cell-free therapies.It has been reported that DCs-EVs are used to induce inflammatory responses in cancer patients, while MSCs-EVs are commonly employed for inflammatory treatment and drug delivery (Shahraki et al. 2023). As such, selecting parental cells that are compatible with the disease type can significantly enhance the therapeutic efficacy. In recent years, preclinical research and clinical trials on cell-free therapies based on endogenous and exogenous exosomes have been actively progressing. In Phase 1 clinical trials, MSCs-exosomes demonstrated favorable safety and therapeutic efficacy in the treatment of complex perianal fistulas, with no adverse effects observed (Pak et al. 2023). Moreover, the United States Food and Drug Administration (FDA) has approved exosome therapy for clinical trials in acute ischemic stroke (Li et al. 2021f). A comprehensive understanding of the biodistribution and excretion pathways of exosomes can assess their stability and persistence, thereby providing critical insights into the safety of exosomes-based therapies. Accordingly, rigorous experiments and animal studies are essential to determine dosage regimens. The most effective therapeutic dosage range can be identified through systematic evaluation of EVs’ biological effects and toxicological responses at varying dosages, achieving a balance between treatment efficacy and safety in clinical trials. Moreover, long-term monitoring of adverse reactions of EV therapy to the human body is crucial. Prompt detection and management of any adverse reactions can effectively ensure the safety and effectiveness of the treatment, bringing more benefits to patients.

**Fig. 2:**
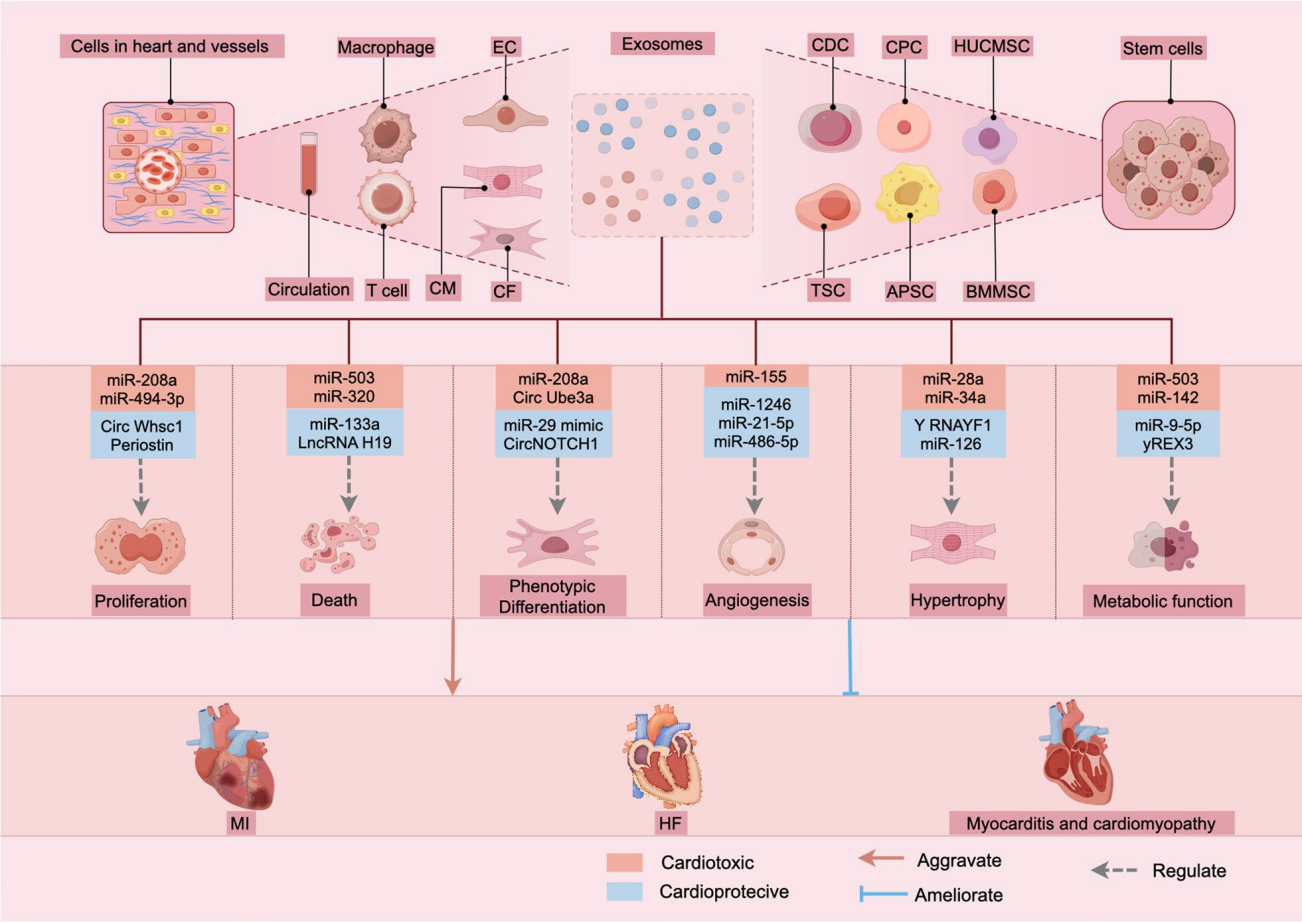
The roles of EV and the potential of cell-Free therapy in heart disease. Extracellular vesicles derived from cardiac resident cells and circulating sources target receptor cells through intercellular communication, influencing their biological functions and thereby affecting the progression of heart disease. Exosomes from stem cells are loaded with cardioprotective molecules, effectively mitigating the damage caused by heart disease, and hold significant promise as a cell-free therapeutic approach. *ASC* adipose-derived stem cell, *BMMSC* bone marrow mesenchymal stem cell, *TSC* trophoblast stem cell, *CDC* cardiosphere-derived cell, *CPC* cardiac progenitor cell, *HUCMSC* human umbilical cord mesenchymal stem cells

**Fig. 3:**
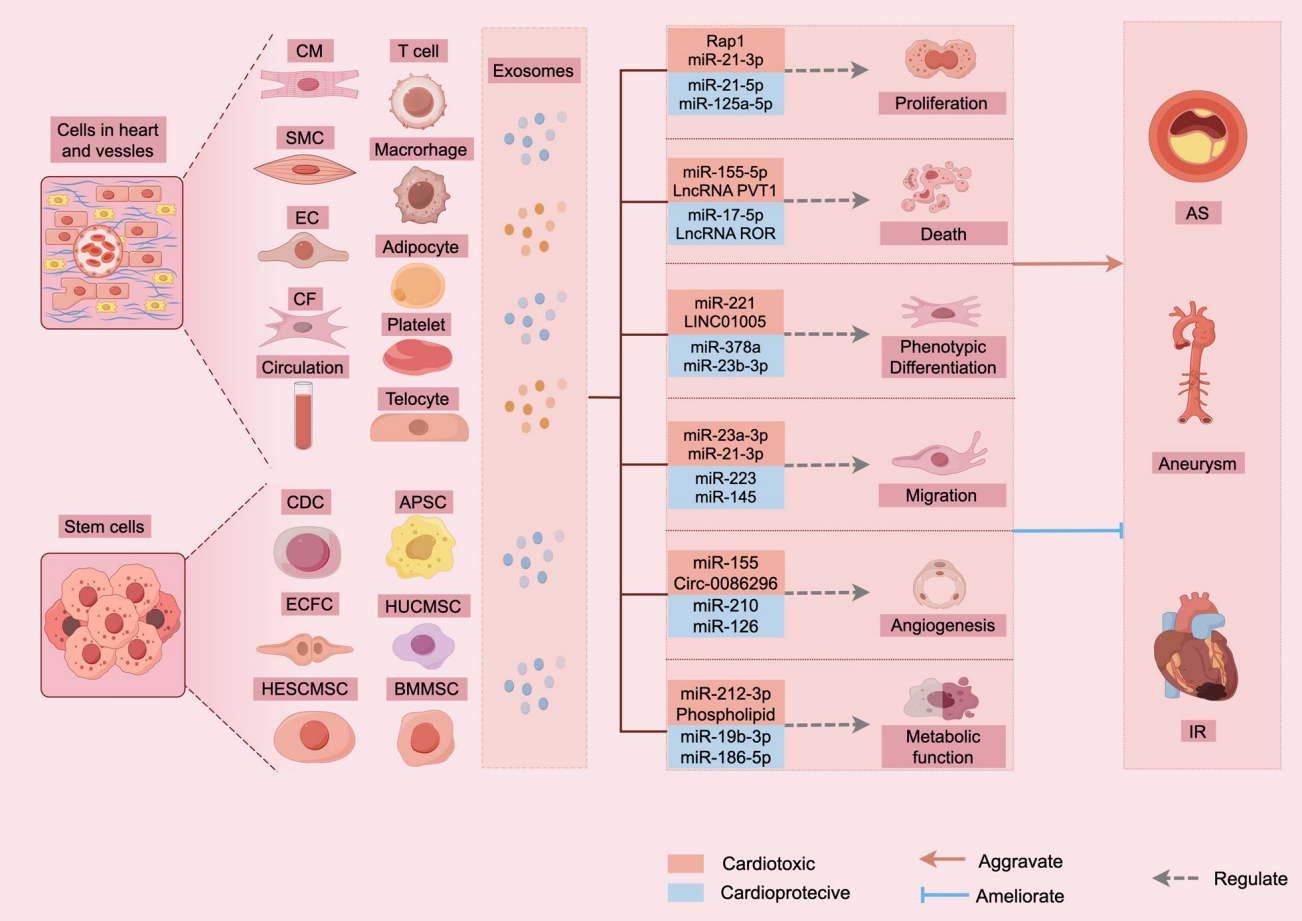
The roles of EV and the potential of cell-Free therapy in vascular disease. Extracellular vesicles, originating from both cardiac resident cells and circulating sources, mediate intercellular communication through interactions with target cells, thereby modulating their biological behavior and influencing the progression of vascular disease. Exosomes derived from stem cells are enriched with cardioprotective molecules, which effectively mitigate the adverse effects of vascular disease, offering a promising cell-free therapeutic strategy. *ECFC* endothelial colony-forming cell, *HESCMSC* human embryonic stem cell derived MSC

Over the past few decades, significant advancements have been made in understanding the biogenesis, structure, and functions of EVs, particularly their role in intercellular communication within the pathophysiology of CVDs (Figs. 2 and 3), as well as their potential in the diagnosis and treatment of CVDs (Table 9). Currently, clinical research on exosomes primarily focuses on biomarkers, cell-free therapies, drug delivery systems, and cancer vaccines. However, to date, no exosome-based drug for CVDs has been approved for market, nor has any relevant biomarker successfully achieved clinical translation due to issues such as poor standardization in exosome production, inadequate targeting precision, and complex underlying mechanisms (Rezaie et al. 2022), indicating that the medical application of exosomes remains at a distance from clinical translation.The therapeutic potential of EVs for drug delivery in cardiovascular diseases largely depends on overcoming specific technical challenges, including advanced separation techniques, cardiac targeting, and *in vivo* metabolism. The lack of effective targeting for damaged myocardium represents a limitation of EVs in cardiovascular therapy and restricts their clinical application. Although studies tracking EVs indicate that their delivery to the myocardium results in higher retention within the heart compared to delivery via coronary or intravenous routes, such methods are often undesirable due to the associated risks of invasive procedures (de Abreu et al., 2020; Gallet et al., 2017). Research has demonstrated that engineered EVs modified with cardiac-targeting titanium can achieve targeted delivery of gene-editing molecules to the heart, significantly improving outcomes in myocardial infarction; however, studies on *in vivo* gene-editing efficiency have yet to be conducted (Mun et al., 2024). Future efforts should focus on interdisciplinary collaboration (such as materials science, imaging, and computational biology) to address these challenges while also establishing a framework for the efficacy evaluation of cardiac-specific exosomes. Overcoming these challenges, an exosome-based cell-free therapeutic strategy is expected to transition from the laboratory to clinical practice.

**Table 9:** Therapeutic potential strategies of stem cells in CVD

Origin	Cargo	Target cell	Model	Target gene/pathway	Function	Refs.
ASC	miR-210	HUVEC, CM	Rat MI,Rat I/R	PTP1B/DAPK1, EFNA3	Cell apoptosis↓, angiogenesis↑, cardiac function↑	Song et al. (2021)
ASC	miRs	CM	Mice TAC	Unknow	CM hypertrophy↓, cardiac function↑	Nakamura et al. (2020)
ASC	miR-17-5p	Macrophage	Mice AAA	TXNIP	M ɸ pyroptosis↓, inflammation↓, AAA formation↓	Hu et al. (2022a)
ASC	miR-19b-3p	VSMC	Mice AAA	MST4	inflammation↓, mitochondrial fission↓, VSMC senescence↓, AAA formation↓	Zhang et al. (2023e)
BMMSC	LncRNA H19	CM, HUVEC, CF	Rat MI	miR675/VEGF/ICAM-1in EC	Cell apoptosis↓, angiogenesis↑, inflammation↓, cardiac function↑	Huang et al. (2020)
BMMSC	miR-486-5p	CF	Monkey and mice MI	MMP19-VEGFA	Angiogenesis↑, cardiac function↑, infarct size↓	Li et al. (2021e)
BMMSC	Unknow	HUVEC	Rat MI	TFEB	EC proliferation↑, angiogenesis↑, autophagy↑, cardiac function↑	Liu et al. (2020a)
BMMSC	miR-125-a-5p	Macrophage, CF, EC	Mice I/R, Swine I/R	KLF13, TGFBR1, DAAM1	Inflammation↓, angiogenesis↑, apoptosis↓, cardiac function↑	Gao et al. (2023)
BMMSC	miR-455-3p	CM	Rat I/R	MEKK1/MKK4/JNK	CM apoptosis↓, autophagy↓	Wang and Shen (2022)
BMMSC	miR-133a	CM	Rat VM	MAML-1	CM apoptosis↓, fibrosis↓, cardiac function↑	Li et al. (2021c)
BMMSC	Unknow	Unknow	Mice diabetes	Unknow	CM apoptosis↓, inflammation↓, fibrosis↓, CM hypertrophy↓, cardiac function↑	(Banerjee and Singla 2024)
BMMSC	circNOTCH1	CF, macrophage	Mice diabetes	NOTCH	CF apoptosis↓, M2ɸ polarization↑, inflammation↓, cardiac function↑	Zhen et al. (2024)
BMMSC	LncRNA NEAT1	CM	Doxo-mice	miR-221-3p	CM senescence↓, apoptosis↓, cardiac function↑	Zhuang et al. (2020)
BMMSC	Unknow	Mast cell	Mice IA	Unknow	Mast cell activation↓, inflammation↓, intracranial aneurysms stability↑	Liu et al. (2016)
BMMSC	miR-23b-3p	Treg cell	Rabbit IA	KLF5	Maintain Th17/Treg balance, pathologic remodeling↓, aneurysm formation↓	Sun et al. (2020b)
TSC	let-7i	CM	Doxo-mice	YAP1/Hippo	CM apoptosis↓, cardiac function↑	Ni et al. (2020)
CDC	hsa-miR-4488	CM	Mice ACM	NF-kB	CM death↓, inflammation↓, cardiac function↑	Lin et al. (2021)
CDC	YF1	CM	Mice HCM	Unknow	CM hypertrophy↓, inflammation↓, cardiac function↑	Huang et al. (2021)
CDC	yREX3	Macrophage	Rat MI	Pick1	Macrophage efferocytosis↑	Ciullo et al. (2024)
CDC	miRs	CM	Swine DCM	TRAF6/SMAD4/FOS	CM proliferation↑, angiogenesis↑, inflammation↓, cardiac function↑	(Hirai et al. 2020)
CPC	periostin	CM	Rat MI	FAK	CM proliferation↑, cardiac function↑	Balbi et al. (2021)
CPC	Unknow	CM	Rat VM	Akt/mTOR, BcL-2/caspase, 4EPB1	CM apoptosis↓, CVB3↓	Li et al. (2019b)
CPC	miR-126	CEC	Rat I/R	Unknow	Angiogenesis↑, fibrosis↓, hypertrophy↓, cardiac function↑	Bheri et al. (2023)
ECFC	miR-21-5p	HCMEC	Rat AS	SIPL1A2	EC proliferation↑, migration↑, and tube formation↑, autophagy↑	Ke et al. (2022)
HESCMSC	Unknow	CM	Mice I/R	PI3K/Akt pathway	ATP↑, CM apoptosis↓, inflammation↓, cardiac function↑	Arslan et al. (2013)
HUCMSC	miR-29 mimic	CF	Mice MI	TGFβ/Smad3	Differentiation to myo-CF↓, inflammation↓, infarct size↓, cardiac function↑	Yuan et al. (2023)
HUCMSC	miR-1246	HUVEC, CM	Mice LAD	PRSS23	Angiogenesis↑, CM apoptosis↓, cardiac function↑	Wang et al. (2021c)
HUCMSC	miR-145	HUVEC	Mice AS	JAM-A	EC migration↓ plaque formation↓	Yang et al. (2021)
HUCMSC	Unknow	CM	Mice VM	AMPK/mTOR	CM apoptosis↓, autophagy↑, inflammation↓, cardiac function↑	Gu et al. (2020)
HUCMSC	Unknow	Unknow	Rat diabetes	AMPK-ULK1	Autophagy↓, fibrosis↓, CM hypertrophy↓, cardiac function↑	Zhang et al. (2022b)
HUCMSC	miR-147	Macrophage	Mice AAA	Unknow	Mɸ activation↓, immune cell infiltration↓, AAA formation↓	Spinosa et al. (2018)
HUCMSC	Unknow	HASMC	Mice AAA	PI3K/AKT	SMC ferroptosis↓, NETosis↓, AAA formation↓	Chen et al. (2023)
iPSC-CM	miR-106a-363 cluster	CM	Mice MI	Jag1-Notch3-Hes1	CM proliferation↑, cardiac function↑	Jung et al. (2021)
iPSC-CPC	miRs	HUVEC, CM	Mice MI	Unknow	Angiogenesis↑, CM apoptosis↓, cardiac function↑	El Harane et al. (2018)
iPSC-MSC	miR-9-5p	CM	Doxo-mice	VPO1/ERK	CM senescence↓, cardiac function↑	Zheng et al. (2024)
MECSMSC	miR-199a-3p, miR-424-5p, miR-21-5p	CM	Doxo-mice	Akt-Sp1/p53	CM apoptosis↓, cardiac function↑	Lee et al. (2021)

*ASC* adipose-derived stem cell, *BMMSC* bone marrow mesenchymal stem cell, *BMSC* bone marrow stromal cell, *TSC* trophoblast stem cell, *CDC* cardiosphere-derived cell, *CPC* cardiac progenitor cell, *CEC* cardiac endothelial cell, *ECFC* endothelial colony-forming cell, *hiPSC* human induced pluripotent stem cell, *HUCMSC* human umbilical cord mesenchymal stem cells, *HESCMSC* human embryonic stem cell derived MSC, *MECSMSC* murine embryonic mesenchymal stem cell, *ACM* arrhythmogenic cardiomyopathy, *HCM* hypertrophic cardiomyopathy, *DCM* dilated cardiomyopathy, *NET* neutrophil extracellular trap, *PBMNC* peripheral blood mononuclear cell, *IA* intracranial aneurysm

## Conclusions

This review comprehensively summarizes the latest research on EVs in CVDs, highlighting their role, potential as biomarkers, prospects for cell therapy, and current challenges. It offers novel insights into the pathophysiology of CVDs, as well as diagnostic, therapeutic strategies, and prognostic assessments. Additionally, it provides directions for future research on EVs and predicts cell-free therapy as a crucial method for treating CVDs, potentially improving outcomes and enhancing patient quality of life.

## Data Availability

No datasets were generated or analysed during the current study.

## Abbreviations

CVDs: Cardiovascular diseases
EVs: Extracellular vesicles
MI: Myocardial infarction
I/R: Ischemia–reperfusion
HF: Heart failure
AS: Atherosclerosis
ESEs: Early sorting endosomes
LSEs: Late sorting endosomes
ILVs: Multiple intraluminal vesicles
MVB: Multivesicular body
ECs: Endothelial cells
CMs: Cardiomyocytes
DAMPs: Danger-associated molecular patterns
HMGB-1: High mobility group box 1
NOS: Nitric oxide synthase
CF: Cardiac fibroblast
LVEF: Left ventricular ejection fraction
MSC: Mesenchymal stem cells
CPC: Cardiac progenitor cells
Ox-LDL: Oxidized low-density lipoprotein
VSMC: Vascular smooth muscle cells
VM: Viral myocarditis
AKT/mTOR: Protein kinase B/mammalian target of rapamycin
DCM: Dilated cardiomyopathy
AAA: Abdominal aortic aneurysm
ADSC: Adipose stem cell
